# Expanding the empirical study of virtual reality beyond empathy to compassion, moral reasoning, and moral foundations

**DOI:** 10.3389/fpsyg.2024.1402754

**Published:** 2024-06-25

**Authors:** Dennis W. Dunivan, Paula Mann, Dale Collins, Dennis P. Wittmer

**Affiliations:** Daniels College of Business, University of Denver, Denver, CO, United States

**Keywords:** moral reasoning, moral foundations, empathy, compassion, virtual reality, moral psychology, experiential learning

## Abstract

This study utilizes a controlled experimental design to investigate the influence of a virtual reality experience on empathy, compassion, moral reasoning, and moral foundations. With continued debate and mixed results from previous studies attempting to show relationships between virtual reality and empathy, this study takes advantage of the technology for its ability to provide a consistent, repeatable experience, broadening the scope of analysis beyond empathy. A systematic literature review identified the most widely used and validated moral psychology assessments for the constructs, and these assessments were administered before and after the virtual reality experience. The study is comprised of two pre-post experiments with student participants from a university in the United States. The first experiment investigated change in empathy and moral foundations among 44 participants, and the second investigated change in compassion and moral reasoning among 69 participants. The results showed no significant change in empathy nor compassion, but significant change in moral reasoning from personal interest to post-conventional stages, and significant increase in the Care/harm factor of moral foundations. By testing four of the primary constructs of moral psychology with the most widely used and validated assessments in controlled experiments, this study attempts to advance our understanding of virtual reality and its potential to influence human morality. It also raises questions about our self-reported assessment tools and provides possible new insights for the constructs examined.

## 1 Introduction

Recent advances in virtual reality (VR) technology and content allow the investigation of simulated moral actions and events in visually immersive environments. VR provides the opportunity to simulate real-life situations, including social situations that can simultaneously trigger the body and the brain (Alcaniz et al., 2018). VR has been proposed as a promising pedagogical tool for education, enabling experiential learning in real-world scenarios that would otherwise be inaccessible or limited by costs, geographic distance, and safety. In VR, a student can be immersed into a refugee camp halfway around the world, experience an underwater dive through an oceanic reef that has been saved by the collective efforts of commercial fisherman, or walk in the shoes of a person from another race, culture, or socio-economic group.

Sholihin et al. (2020) found that VR-based media enhanced learning processes by making them more motivating and interesting, which led to increased ethical efficiency and self-efficacy in students. Herrera et al. (2018) found participants in a VR condition offered more compassionate support of affordable housing for the homeless than participants in less immersive conditions. VR has been touted as the “ultimate empathy machine” (Milk, 2015). However, in their review of literature from 2015 to 2020, which examined quantitative links of VR with empathy, Villalba et al. (2021) found a lack of rigorous, empirical scientific evidence to prove unequivocally that VR is a vehicle for developing empathy, as many scholars, human rights organizations, and educators suggest.

In the article, Disrupting the “empathy machine”: The Power and perils of virtual reality in addressing social issues, Sora-Domenjó (2022) reviewed the literature from the fields of psychology, computer science, embodiment, medicine and virtual reality, revealing little empirical evidence of a correlation between VR exposure and an increase in empathy that motivates pro-social behavior. Sora-Domenjó (2022) states that efforts must be made to disrupt the VR empathy-model. This study seeks to contribute to this effort. It posits that what researchers, educators, and proponents of VR have been measuring in support of motivating pro-social behavior, might be missing an opportunity by focusing too much on the VR empathy-model and not considering other constructs that may provide useful knowledge and opportunities. With the results of this study showing links between a VR experience, moral reasoning and moral foundations, but not empathy nor compassion, we open up a new set of questions about how we define these constructs, how we measure them and how we might possibly incorporate them into efforts supporting pro-social behavior.

The research began with a systematic literature review to identify and evaluate potential constructs to be tested. These constructs and their assessments were identified and evaluated for relevance to ethically and morally based decision making. The assessments of these constructs were chosen for strong psychometric properties, validity, and broad use among scholars, encouraging the development of consistent measures to be used in future studies. These assessments include the IRI (Davis, 1983) for empathy, the DIT-2 (Rest, 1986a) for moral reasoning, the MFQ30 (Haidt, 2012a,b) for moral foundations, and the Pommier et al. (2020) compassion scale (CS). The pre-post experimental design also included control assessments for political orientation and social desirability.

Virtual reality has been used as a methodological tool to study social behavior for at least two decades (Blascovich et al., 2002). Beyond its immersive capabilities, VR provides an instrument to help overcome some of the challenges of psychological experiments, including reproducibility, ecological validity, and experimental control (Pan and Hamilton, 2018). This study takes advantage of these attributes by testing how the same VR experience possibly influences change in a participant’s empathy, compassion, moral reasoning, and moral foundations in two controlled, pre-post experiments. The treatment experience was a VR film entitled *The Displaced*, which depicts the dystopia of refugee children, and the control experience was a VR film documentary of the history of cinema called *Kinoscope*. The next section describes the theoretical background of the constructs along with the logic used for hypothesis development.

## 2 Theoretical background of hypothesized constructs

### 2.1 The possible influence of virtual reality on empathy—mixed results

To understand VR’s possible influence on empathy, we must begin with clearly defined definitions along with realistic expectations for the capabilities of the assessment tools. However, a review of the literature on empathy yields multiple definitions, including meanings with various cognitive, affective, and behavioral components. In their meta-analysis to review the concept of empathy, Cuff et al. (2016) used a snowballing procedure to identify empathy definitions in the literature from key articles and found 43 distinct definitions. Empathy has been studied across disciplines, including philosophy, development, etiology, cognitive and social psychology, and neuroscience (Zaki, 2014). Batson and Ahmad (2009) identify four psychological states called empathy. The four psychological states are divided between cognitive/perceptual states (1) imagine-self perspective (2) imagine-other perspective and affective/emotional states (3) emotion matching and (4) empathetic concern. Cognitive empathy, or Theory of Mind, is the ability to understand and represent another’s feelings (Blair, 2005). Affective empathy is concerned with the ability to understand another person’s emotions and respond appropriately. Oliveira-Silva and Gonçalves (2011) define empathy as “The capacities to resonate with another person’s emotions, understand his/her thoughts and feelings, separate our own thoughts and emotions from those of the observed and responding with the appropriate prosocial and helpful behavior.” Prosocial behavior is voluntary behavior intended to benefit another (Mussen and Eisenberg-Berg, 1977).

When speaking about empathy, it is important to discuss what empathy is not because it is often commingled with other constructs. The related concepts of compathy, mimpathy, sympathy, transpathy, and unipathy are discussed in the literature in relation to empathy (Cuff et al., 2016). Ickes (2003) makes a clear distinction between the emotions and notes that each construct differs in its degree of cognitive representation of the target’s emotional state, degree of emotion sharing, and the degree to which a self and other distinctions are maintained. Of the linked constructs, sympathy appears to be the most frequently discussed in comparison to empathy. Sympathy is meaningfully different from empathy. Whereas empathy involves cognitively taking the perspective of another, sympathy involves the other-oriented desire for the other person to feel better (Eisenberg and Fabes, 1990). Sympathy is not the same as feeling what the other person feels. Hein and Singer (2008, p. 157) in their review of empathy from the perspective of neuroscience, describe the difference between empathy and sympathy as “feeling as and feeling for the other.”

With a conceptual definition of empathy in mind, it is necessary to answer whether empathy can be developed and, if so, what the value of inducing empathy might be. Humans have the capacity to control and regulate their emotions through various conscious and unconscious strategies (Williams and Wood, 2010). Empirical research shows that imagining what it would be like to be someone else (perspective-taking) can be a potent mechanism to promote empathy and motivate prosocial behaviors (Coke et al., 1978; Batson et al., 1988; Eisenberg and Fabes, 1990; Batson and Ahmad, 2009). A 2011 study found that dispositional, empathetic concern predicted prosocial intentions and behavior via the mediation of autonomous motivation, e.g., motivated by interest, enjoyment, and personal values (Pavey et al., 2012). Further, if empathy evokes autonomous motivation to help others, this will support the use of empathy-arousing media such as VR experiences to promote altruistic behaviors.

In her review of how immersive journalism can possibly enhance empathy, Sánchez Laws (2020) describes the neurological basis for how empathy might lead to pro-social behavior through VR. She states that when we can directly observe others in pain, our somatosensory cortex is activated, thus we use the same brain area involved in our own sensory experience of pain to perceive someone else’s pain. Both the evolutionary and the neuroscientific perspectives point to the possibility that we are hardwired to use empathy as a mechanism for action in the world. Thus, empathy could possibly be a mechanism through which we gather information to cooperate with others.

Virtual reality experiences place users in novel environments, showing them what it would be like to experience a specific situation from someone else’s perspective. One goal of VR storytelling is to stimulate emotions that will influence action (Shin, 2018). Unique to VR and what makes it different from more traditional media (e.g., radio, TV, and movies) is the immersive environment that can offer visual, auditory, and tactile stimuli. VR allows the participant to interact actively with their virtual ecology and experience life-like surroundings from someone else’s perspective. The perspective-taking aspect of VR makes it an intriguing tool for inducing emotion, which has led to the speculation that it can specifically increase empathy, to influence prosocial behaviors.

A recent study found VR perspective-taking tasks to potentially be more effective at improving attitudes toward the homeless and motivating prosocial behaviors than less immersive perspective-taking tasks (Herrera et al., 2018). In order to examine perspective-taking and empathy through virtual reality, Seinfeld et al. (2018) had convicted male abusers inhabit the body of an abused woman to induce a “full body ownership illusion.” After being embodied in a female victim, offenders improved their ability to recognize fearful female faces and reduced their bias toward recognizing fearful faces as happy. The study demonstrated that changing the perspective of an aggressive population through immersive virtual reality can modify socio-perceptual processes such as emotion recognition.

Yoo and Drumwright (2018) used two different media (VR and a tablet) to solicit donations for a not-for-profit organization and found donation intention, perceived vividness, perceived interactivity, and social presence were all significantly greater with VR. Another experiment utilized a VR experience to place participants in the home of a young Syrian refugee and showed that the VR experience led to a higher level of two dimensions of empathy, empathic perspective-taking and empathic concern (Schutte and Stilinović, 2017).

Lastly, there is some evidence the VR perspective-taking experiences may be more durable than other perspective-taking tasks (Herrera et al., 2018). Based on the above evidence, there is sufficient theoretical and empirical justification to warrant further exploration into VR’s impact on individual empathy, while at the same time recognizing the mixed results and limitations found in the reviews of Villalba et al. (2021) and Sora-Domenjó (2022).

### 2.2 Differentiation of empathy and compassion

To begin framing the research question and hypotheses of this study, we start with an attempt to more clearly define and differentiate empathy, sympathy and compassion. Although the precise definitions of these constructs are many-times debated (Cuff et al., 2016; Bloom, 2017; Hall and Schwartz, 2019), there is general agreement among scholars that *sympathy* can be described as one person *understanding* what another person is feeling, *empathy* can be described as one person *feeling* what another person is feeling, and *compassion* is the *desire to relieve the suffering* of another.

Bloom (2017) offered the distinction that *empathy* refers more generally to our ability to take the perspective of and feel the emotions of another person, whereas *compassion* is when those feelings and thoughts include the motivation to help. He made the theoretical argument that compassion is distinct from empathy in its neural instantiation and behavioral consequences, stating that compassion is a better prod to moral action. In their article, “The Neuroscience of Empathy and Compassion in Pro-social Behavior,” Stevens and Taber (2021) showed how functional neuroimaging research can help us see how the components of sympathy, empathy, and compassion are associated with distinct brain processes marked by co-activation among brain regions.

In the boundaries of this study, we contribute to the discussion regarding the distinction between *empathy* and *compassion*. Even though VR has been described as the “ultimate empathy machine,” as it allows people to viscerally experience another person’s point of view (Milk, 2015), empirical evidence of a direct relationship between VR and empathy is still inconclusive. The review by Villalba et al. (2021), which summarized, critiqued, and sought to advance VR as a pedagogical tool, found research on integrating the technology into educational programs to be promising. However, they reported a lack of rigorous, empirical evidence of direct relationships between VR and the development of empathy. They pointed to the inadequate quantification of empathy in most of the studies they reviewed as a potential challenge and recommended the Interpersonal Reactivity Index (IRI), created by Davis in 1983, as a potentially useful tool to remedy this. The IRI was used by Herrera et al. (2018) in a study that compared students experiencing homelessness via VR to traditional perspective-taking methods. The results of this study did not find a significant relationship between the VR experience and empathy as measured by the IRI but did find a significant relationship between the VR experience and the motivation of pro-social behaviors; after their VR experiences, participants agreed to sign petitions to help the homeless.

To further our understanding of how one VR experience may influence empathy vs. compassion, the experimental design for this study allows us to analyze assessments of empathy and compassion pre-post of the same VR experience. While theory supports a hypothetical model for direct effects, we also analyze empathy as a mediator for moral foundations and compassion as a mediator of moral reasoning on an exploratory basis. The IRI (Davis, 1983) is used as the assessment of empathy to maintain consistency with the study of Herrera et al. (2018), and also to accept the recommendation of Villalba et al. (2021). While Herrera et al. (2018) used a direct measurement of donations to measure compassion, our study incorporates both a direct measurement of donations and a pre-post assessment of the compassion scale of Pommier et al. (2020).

In their review of compassion assessment scales, Strauss et al. (2016) proposed five elements of compassion extracted from their synthesis of definitions and analysis, which define compassion as a cognitive, affective, and behavioral process. These elements include: (1) recognizing suffering; (2) understanding the universality of suffering in the human experience; (3) feeling empathy for the person suffering and connecting with their distress (i.e., emotional resonance); (4) tolerating uncomfortable feelings aroused in response to the suffering person (e.g., distress, anger, fear) so remaining open to and accepting of the person suffering; and (5) motivation to act to alleviate suffering.

Pommier et al. (2020) proposed that their compassion scale is superior to other compassion scales in meeting the criteria proposed by Strauss et al. (2016), as listed above. They specifically argued that their measurements to assess recognition of common humanity in the experience of suffering more closely address two of the elements of Strauss et al. (2016): (1) recognizing suffering and (2) motivation to alleviate suffering, which distinguishes it from pity. This intricate distinction is important to the current study because pity, as defined by Pommier et al. (2020), fosters a sense of distance and *disconnection*, whereas compassion has *connection* as its core (Strauss et al., 2016; Bloom, 2017; Pommier et al., 2020). This connection is thought to be a possible mechanism proposed in this study as a mediator between the VR experience and moral reasoning.

### 2.3 Moral reasoning and cognitive moral development

The Kohlberg and Kramer’s (1969) theory of moral reasoning has been cited and supported in thousands of studies over the past 60 years and is still prevalent in literature today (Gurley and Dagley, 2021; Villalba et al., 2021). Kohlberg extended Piaget’s (1932) theory by identifying six stages of moral reasoning capability. He adopted and further developed technique of Piaget (1975) of telling stories involving moral dilemmas. In each case, he presented a choice to be considered––for example, a choice between the rights of an authority vs. the needs of a potentially deserving individual who could be viewed as being unfairly treated. For this study, the term *moral reasoning* is used as it relates to Kohlberg’s theory in general and the term *cognitive moral development* (CMD) as it relates to the specific levels and stages identified in his theory. As Trevino (1986) pointed out in a description of the CMD model as a major component of her work on ethical leadership, the emphasis of CMD is on the cognitive decision-making process rather than the decision itself. It is the *process* of reasoning that differentiates a person’s level or stage.

Table 1 shows the levels and stages of CMD proposed by Kohlberg and Kramer (1969) and further defined in his article, “Moral Development: A Review of the Theory” (Kohlberg and Hersh, 1977). The Center for the Study of Ethical Development, which provides moral schema scoring for the DIT-2 analysis of moral reasoning used in this study, reports Kohlberg’s pre-conventional level as *Personal Interest*, the conventional level as *Maintaining Norms,* and the fifth and sixth stages representing societal interests and universal principles as the *Post-Conventional* level. The results of this study are reported using the Center for the Study of Ethical Development’s DIT-2 moral schema scoring labels, which is discussed further in the methods section.

**Table 1:** Stages of cognitive moral development (Kohlberg and Kramer, 1969).

Level of CMD		Stage of CMD
Personal Interest (Pre-Conventional)	Stage 1	Acting to avoid pain and punishment.
	Stage 2	Right action consists of that which satisfies one’s own needs. Reciprocity is a matter of “you scratch my back and I’ll scratch yours,” not of loyalty, gratitude, or justice.
Maintaining Norms (Conventional)	Stage 3	Interpersonal concordance or “good boy/nice girl” orientation. Good behavior is that which pleases others and is approved by them. Conformity to “natural” behavior.
	Stage 4	A “law and order” orientation toward authority and fixed rules. Right behavior consists of doing one’s duty, showing respect for authority, and maintaining social order.
Post-Conventional (Societal/Universal)	Stage 5	Social-contract orientation with utilitarian overtones. Right action defined in terms of individual rights critically examined and agreed upon by the whole society.
	Stage 6	Universal principles of justice and human rights. Respect for human dignity, defined by decisions of logical comprehensiveness, universality, and consistency.

Each level of CMD proposed by Kohlberg and Kramer (1969) contain two corresponding stages. At the *pre-conventional* level, a person is responsive to cultural rules and labels of “good” and “bad,” but they interpret these labels in terms of physical or hedonistic consequences (Kohlberg and Hersh, 1977). In the first stage of CMD, a person acts to avoid punishment––not because something is morally right, but because punishment hurts. In the second stage, a person acts to further their own interest or to satisfy their own needs.

At the *conventional* level, a person strives to maintain the expectations of their family, group, or even nation. This act of conformity and loyalty is thought to be driven by a societal need to maintain order, which in turn benefits the individual. It includes the third and fourth stages of CMD. In the third stage, which is sometimes referred to as “good boy/nice girl” orientation, a person acts to conform to societal norms and is rewarded with approval for this conformity. In the fourth stage, there is an added conformity to law and order. The orientation is toward authority, fixed rules, and the maintenance of social order. Correct behavior is showing respect for authority, but it is still driven by the need for social order for one’s own sake.

At the *post-conventional* level, a person develops an intention to define moral values and principles that have validity and application apart from authority and beyond their identification of their own group (Kohlberg and Hersh, 1977). This highest level of CMD includes the fifth and sixth stages of moral development. The fifth stage is termed *social contract orientation* and has utilitarian overtones. Although it still has a legalistic point of view, which is described in the fourth stage as “law and order,” the fifth stage emphasizes the possibility of changing the law in terms of rational considerations for individual rights. In the fifth stage, rights are a matter of personal values and opinions. The fifth stage is similar to the morality placed on the United States government and constitution (Kohlberg and Hersh, 1977).

The sixth and highest stage of CMD is termed *universal-principled orientation.* Morality is defined by a decision of conscience, which can be influenced by experiences and education (Kohlberg, 1976). The principles of the sixth stage are abstract and ethical. These universal principles align more with what Western religions refer to as the Golden Rule or what Eastern religions refer to as Karma. Kohlberg (1971) states, these are universal principles of justice, of the reciprocity and equality of human rights, and of respect for the dignity of human beings as individual persons.

Moral development does not simply represent an increasing knowledge of cultural values leading to ethical relativity. According to Piaget (1932), it represents a transformation that occurs in a person’s form or structure of thought. Although the *content* of values can vary from culture to culture, the *structure* of an individual’s moral judgment is universal across cultures. A theoretical viewpoint being considered in this study is that immersive VR experiences can allow viewers to directly experience the perspective or role of another person and the context or environment surrounding that perspective or role. This study analyzes how treatment and control VR experiences impact a person’s CMD with pre-post measurements of moral reasoning. Research suggests that people can progress to higher stages of moral reasoning through their experiences (Miller et al., 1980; Trevino et al., 2000; Gurley and Dagley, 2021). A possible contribution of this study is to help enhance the methods used by educators in this pursuit, to further bridge the gap between the theory of CMD and experiential education, leading to more effective pedagogical methods.

### 2.4 Moral foundations theory

One of the primary questions debated by scholars of moral psychology is how much of human morality is genetic, how much is self-constructed, and how much is influenced by external factors like parents, society or other experiences (Graham et al., 2013). Stages of Moral Development of Kohlberg and Kramer (1969) and James Rest’s Four Component Model of moral reasoning (1974) provided the theoretical foundation to measure a person’s level of moral reasoning. These accepted models have been applied to research in psychology, education, medicine, business, and many other disciplines. However, in the past 20 years, Jonathan Haidt’s moral foundations theory has challenged Kohlberg, Piaget, and even the foundations of moral thought dating back to Plato, with evidence demonstrating that morality is constructed more by intuition than by reasoning.

Moral foundations theory (MFT) is described by Graham et al. (2013) as descriptive vs. normative. It originates from the notion that one construct or one foundation, like moral reasoning (Kohlberg and Kramer, 1969), or sensitivity to harm (Gray et al., 2012), or generalized human welfare (Harris, 2011), are not adequate to explain the complexities of morality. Haidt questions Kohlberg’s dismissal of Aristotle’s pluralistic view of morality as a “bag of virtues,” and Haidt embraces plurality, proposing that this approach has led MFT to discoveries that were previously missed by monist theories.

There are four components or claims used to summarize MFT (Graham et al., 2013). Nativism articulates a “first draft” of the moral mind, in which nature provides the first draft, and then experience revises it. Cultural Learning is the process whereby the first draft of the moral mind is edited during development within a particular culture. For instance, in some cultures, eating a dog might be considered immoral, but dog meat may be considered good cuisine in other cultures. With the concept of Intuitionism, personal intuition comes first, and strategic reasoning comes second. The basic premise of this claim is that people use reasoning to justify their moral intuition. These processes are based on Haidt’s (2001) Social Intuitionist Model (SIM). The SIM incorporates System 1 thinking (Stanovich and West, 2000; Kahneman, 2011) in which moral evaluations occur rapidly and automatically and System 2 thinking in which moral evaluations are more effortful and deliberate.

Along with his colleagues in Moral Foundations Theory: The Pragmatic Validity of Moral Pluralism (Graham et al., 2013), Haidt proposed five original foundations of intuitive ethics. These foundations and associated descriptions are as follows.

The Care/harm foundation is linked to a person’s innate functional system to automatically connect perceptions of suffering from motivations of care to protect children. The original triggers of Care/harm are visual and auditory signs of suffering and distress. Studies have also now shown these emotions can include anger toward a perpetrator of harm. These moral emotions are not just realized at the individual level, but also at the societal level, where people engage in “gossip” or discussions about people who are not physically present, and these discussions may include moral evaluations of those parties (Dunbar, 1996).

The Fairness/cheating foundation evolved from the advantage some social animals gained from having minds that were sensitive to evidence of cheating and cooperation over those who did not possess this ability (Trivers, 1971). The original triggers of Fairness/cheating were with one’s own direct relationships, including family and tribe. These have since grown to include social media groups and even mechanical things like vending machines that might cheat someone out of their bag of chips.

The Loyalty/betrayal foundation recognizes the advantages gained by individuals and groups whose minds have greater organizational ability in advance of an experience (Sherif and Sherif, 2010). This ability helped some individual leaders and groups to control or even eliminate other less capable individuals and groups. A current example of this includes sports fandom and brand loyalty.

The Authority/subversion foundation is linked somewhat to Loyalty/betrayal such that those individuals whose minds are structured in advance of experience to navigate hierarchies of authority (including psychological, social, and physical power) will gain advantages over those who fail to perceive or react to these complex social interactions. These can involve smaller individual groups like sports teams, larger groups, institutions, or even countries where law, courts, police, government officials, and political leaders are instilled.

The Sanctity/degradation foundation evolved from our need to avoid risks from pathogens and parasites as we moved out of the trees and into larger and denser groups or tribes. The emotion of disgust is thought to be an adaption related to this foundation (Oaten et al., 2009). Individuals with minds that were structured in advance of experience had the ability to develop a more effective “behavioral immune system.” They were not simply reacting to taste and smell but also past knowledge of danger. Self-preservation led to cultural customs involving diet, hygiene, and sexual practices linked to morality.

Haidt and his colleagues are careful to note they do not believe these are the only foundations of morality. They state that while MFT’s origins were in anthropology and evolutionary theory, its development has been connected with the creation and validation of psychological methods to test its claims. They acknowledge the current and future development of MFT to be a method-theory co-evolution (Graham et al., 2013).

## 3 Research overview

This study includes two controlled experiments, which analyzed the change in pre-post assessments of empathy, compassion, moral reasoning, and moral foundations before and after participants viewed either a treatment or control VR film experience. The treatment experience was a VR film entitled *The Displaced*, which depicts the dystopia of refugee children, and the control experience was a VR film documentary of the history of cinema called *Kinoscope*. A review of potential VR experiences was conducted and is described below along with descriptions of both the treatment and control.

Participants included a total sample of 113 undergraduate business students from a university in the western United States. The study was introduced to the students via zoom conference calls and in class with follow-up emails for pre-post surveys. The purpose of the study was not communicated, but the virtual reality component of the experiment was described and possible implications for the metaverse were mentioned, which aligned with the content of the student’s business courses.

In phase 1 of both experiments, the participants completed a questionnaire consisting of three sections. Both experiments included all demographic information necessary for the control variables. Experiment 1 included the DIT-2 (Rest et al., 1999) for moral reasoning and the compassion scale (CS) of Pommier et al. (2020). Experiment 2 included the IRI (Davis, 1983) for empathy and the MFQ30 (Haidt, 2012a,b) for moral foundations. For phase 1, the participants were asked to complete all three sections. Each participant was assigned a unique reference number by Qualtrics that allowed synchronizing data collection from phase 1 with phase 2 of the experiments.

Approximately 2 weeks after the pre-assessment, participants were guided through phase 2 of the study at the CiBiC lab, where they were randomly selected to view either the treatment VR film or control VR film. Immediately following the film, the participants completed the post-assessment survey at computer stations set up in the office of the lab and managed by a lab assistant. These surveys included the coinciding construct assessments taken approximately 2 weeks before the VR experience so the pre-post results could be analyzed. An expedited review from the University of Denver Internal Review Board (IRB) was approved prior to the start of the study, and an implied consent form was signed by all participants prior to the pre-assessment survey. The experimental portions of the study were conducted over 3-week windows from pre-assessment to treatment to post-assessment. To control for outside factors that might have possibly influenced participants during these three-week windows, both treatment and control VR experiences were tested simultaneously within the same sample of participants.

### 3.1 Virtual reality treatment and control variable selection process

The study makes use of two VR film experiences, selected from evaluations of more than 25 publicly available candidates. Eleven VR films were selected by the lead author based on production quality, length of film, and possible emotional stimulus related to the constructs to be tested. Three members of the research team then evaluated and selected three films based on the criteria of, (1) production quality, (2) length of film, (3) story believability, (4) probability of the film to influence empathy or compassion, (5) probability of the film to influence moral reasoning, (6) probability of the film to influence moral foundations, and (7) a manipulation check for awe. The manipulation check for awe was conducted with 12 graduate students using an awe scale developed by Yaden et al. (2019).

*The Displaced* refugee film was chosen as the study’s independent variable because it was expected to have highest possible influence on the constructs of interest based on the criteria. *Kinoscope* was chosen as the control VR film because it was expected to elicit low influence on the constructs.

### 3.2 Virtual reality treatment and control variable descriptions

The Displaced VR film, Solomon and Imraan (2015) was used as the independent variable (IV) in both Experiment 1 and Experiment 2. It was produced by within and created as part of a New York Times multimedia documentary project designed to elicit support for the international refugee crisis. In the film, the viewer is immersed into the stories of an 11-year-old refugee boy from eastern Ukraine named Oleg, a 12-year-old Syrian girl named Hana, and a 9-year-old South Sudanese boy named Chuol. The children speak about how they escaped war zones, their memories of their homes, and their hope for the future. The prediction of the study was that *The Displaced* would possibly influence among participant viewers an increase in empathy, compassion, moral reasoning, and the Care/harm factor of moral foundations as measured by the pre-post assessments. Possible mediation between the constructs was also analyzed.

The control experience for both experiments in the study, a VR film entitled *Kinoscope* (Collin and Leotard, 2017) is a documentary of the history of cinema. It describes the productions of Méliès, Chaplin, Tarantino, and many other producers and directors of famous cinematic productions. *Kinoscope* takes the viewer on a journey through famous scenes captured on film. The prediction was that *Kinoscope* would not influence a change in pre-post measurements of the constructs in the study.

### 3.3 Statistical analysis plan

A statistical power analysis was conducted using G*Power version 3.1.9.7 (Faul et al., 2007) to determine the optimal sample size for the hypotheses in the study. Because no previous studies have tested the dependent and independent variables in relation to each other, Cohen’s (1988) general guidelines for detecting small (*d* = 0.2), medium (*d* = 0.5), and large (*d* = 0.8) effects were used to calculate optimal sample size. Results of the G*Power analysis indicated the required sample size to achieve 80% power for detecting a small effect, at a significance criterion of α = 0.05, was *N* = 156 for a one-tailed *t*-test analyzing the difference between two dependent means (matched pairs). To detect a medium effect size, the optimal sample size is 27, and to detect a large effect size, the optimal sample size is 12. Sample size range to test the hypotheses in the study is *N* = 19 to *N* = 35.

Hypotheses were tested by first calculating the pre-post mean change scores for each of the dependent variables, including the moral reasoning schema variables of personal interest change, maintaining norms change, and postconventional change, along with the compassion scale change for Experiment 1. The pre-post change for the factors of moral foundations and empathy were calculated for Experiment 2. To determine the significance of the pre-post change in mean, a paired sample *t*-test was conducted in SPSS for each set of variables.

Recommendations from Lakens (2022) were followed to maximize the validity of the findings. This included directional hypotheses and utilization of one-tailed, paired sample *t*-tests, which move the Type I error rate to one side of the tail of the distribution, lowering the critical value, and therefore requiring less observations to achieve similar statistical power. To test if mediation occurred while comparing the results of *The Displaced* and *Kinoscope*, simple mediation analysis was performed using PROCESS (Hayes, 2013).

## 4 Experiment 1

### 4.1 Hypothesis development

**Figure 1:**
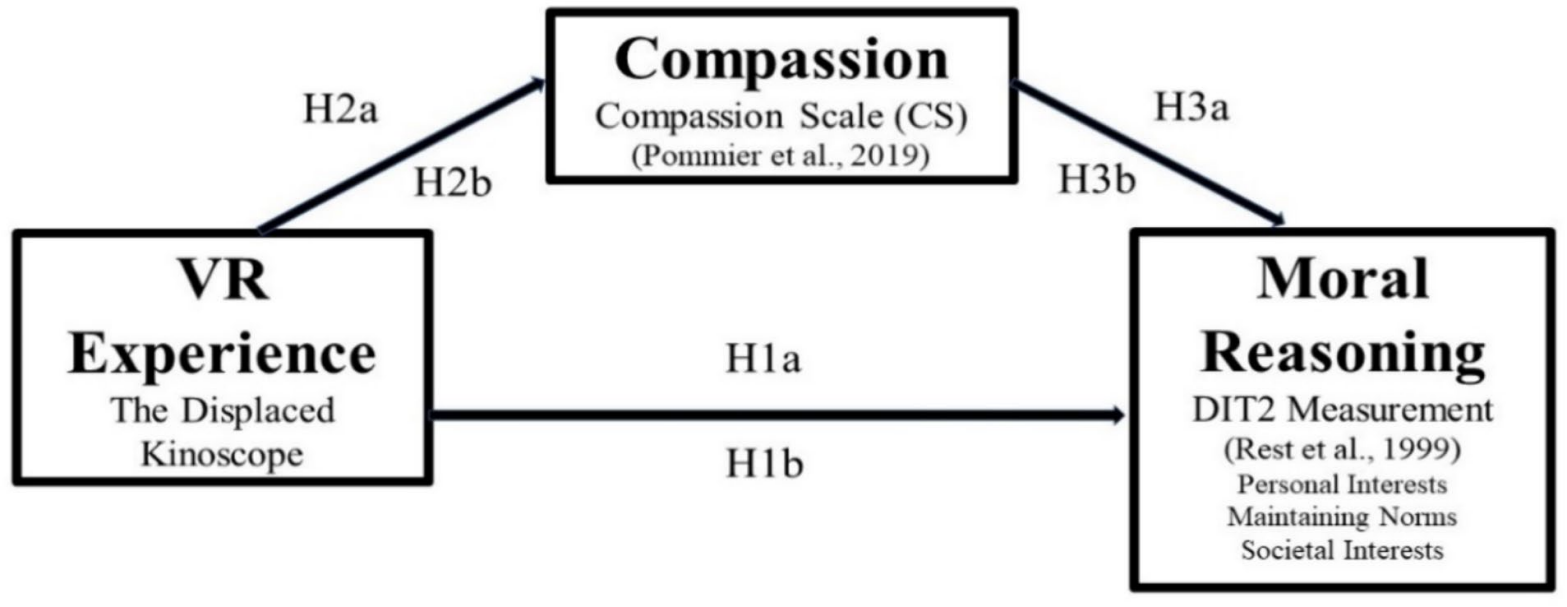
Hypothesized model for Experiment 1.

Hypothesis development for Experiment 1 is based on predicted links among the VR treatment and control experiences described in the previous section, changes in compassion, and changes in moral reasoning as shown in Figure 1.

The experiment includes one overall sample of participants randomly chosen for either the treatment experience or the control experience. *The Displaced* was tested as the treatment experience (H1a), and *Kinoscope* was tested as a control experience (H1b). H1a predicts that *The Displaced* will influence transition of a participant’s moral reasoning from the lower stages of personal interest toward the higher stages of societal interest or postconventional morality (Kohlberg and Hersh, 1977; Bebeau et al., 2008). Although VR experiences have not previously been evaluated for their influence of moral reasoning, this prediction is supported by the findings of studies where other types of experiential learning has influenced higher stages of moral reasoning (Smith et al., 2002; Goralnik and Nelson, 2017).

H1a: The Displaced *virtual reality experience will influence transition of the participant’s level of moral reasoning to a higher stage.*

To control for factors outside the study that may have influenced the results of H1a, the influence of Kinoscope was tested as an experience not expected to influence moral reasoning. The logic of this approach is that if the participant’s stage of moral reasoning in the H1a group significantly transitions to higher stages while the moral reasoning of participants in the H1b group does not significantly transition to higher stages, an assumption can be made that outside influences were not the cause of this transition because they would have influenced both groups. Therefore, H1b predicts that *Kinoscope* will not influence transition of a participant’s moral reasoning from the lower stages of personal interest to the higher stages of societal interest or postconventional morality.

H1b: *The* Kinoscope *virtual reality experience will not influence transition of the participant’s level of moral reasoning to a higher stage.*

The prediction of the second set of hypotheses (H2a and H2b) is that *The Displaced* VR experience will influence compassion, because the participant will feel a desire to help the refugee children depicted in the film. In previous studies, the influence of VR experiences on empathy have been inconclusive (Seinfeld et al., 2018; Villalba et al., 2021; Sora-Domenjó, 2022), but a theoretical argument has been made by Bloom (2017) that compassion is distinct from empathy in its neural instantiation and behavioral consequences. Bloom stated that compassion is a better “prod to moral action.” The second prediction of this study investigates if *The Displaced* increases a participant’s level of compassion, thus providing possible evidence of distinction between compassion tested in our first experiment and empathy tested in our second experiment.

H2a: *Compassion levels will be higher after the participant is immersed into* The Displaced *VR experience.*

In alignment with the methodology used for H1a and H1b, the second prediction is tested among two experimental groups within the same participant sample, to control for outside factors, which might have influenced results during the 3-week period, from pre-assessment to treatment to post-assessment.

H2b: *Compassion levels will not be higher after the participant is immersed into the* Kinoscope *VR experience.*

Based on the results of the first and second predictions, statistical analysis determines the possible mediation influence of compassion on the possible transition of moral reasoning for each of the experimental groups.

H3a: *Compassion will partially mediate the relationship between* The Displaced *VR experience and transition of the participants moral reasoning.*

H3b: *Compassion will not mediate the relationship between the* Kinoscope *VR experience and transition of the participants moral reasoning.*

It is important to note that the purpose of the study and the associated hypotheses are not proposed to compare the direct level of effects between the VR experiences. The study is designed to test if one experience, *The Displaced,* will influence moral reasoning and/or compassion, whereas another experience, *Kinoscope* will not influence moral reasoning and/or compassion. In the future, studies utilizing a larger sample size can help us increase the statistical power necessary to hypothesize and test for possible significant differences in effect between the treatment and control groups. Keeping tight boundaries on the purpose and hypotheses of this foundational study are intentional to increase the validity of the findings with the limited resources and sample size available. Details for the methods of each experiment are described below.

### 4.2 Methods

#### 4.2.1 Participants and procedure

Business students from a university in the Western United States were offered $10.00 for completing all components of the experiment, including the pre-assessment, VR experience, and post-assessment. Although utilizing a student sample is recognized for its possible limitations and overuse in academic research, it is considered appropriate for this study because of the potential implications to integrate findings into pedagogical applications for university course curriculum. To limit the possibility of outside factors and conditions influencing changes in the pre-post results of the dependent variable assessments, a window of 21 days was set for the implementation of the experiment from the participant’s pre-assessment to treatment to post assessment. 69 students completed all phases of the experiment with the random assignment of 34 participants to the treatment group and 35 participants to the control group. Five participants did not pass the reliability checks performed by the Center for the Study of Ethical Development in their analysis of the DIT-2 scores, and the data from these five participants was purged from the overall study sample including all dependent variables and controls.

The research design included dependent variable assessments pre-post of the participants viewing VR experiences in the lab. Qualtrics was used to administer all pre-post assessments, with demographic information collected with the pre-assessment. Approximately 2 weeks after the pre-assessment, participants were guided through phase 2 of the study at the CiBiC lab, where they were randomly selected to view either the IV treatment or control VR experience. Immediately following the experience, the participants completed a post-assessment survey at computer stations set up in the office of the lab and managed by a lab assistant. The lab assistant verified and recorded the VR experience viewed by each participant and provided compensation of $10.00 upon completion of the post-assessment.

#### 4.2.2 Dependent variable and control measurements

The study utilized two highly tested and validated assessment tools to measure moral reasoning and compassion as the dependent variables. The reliability of both assessment tools has been shown with the findings published in top journals of psychology, sociology, education, business, and other disciplines (Davis, 1983; Rest, 1986b; Rest et al., 1999; Trevino et al., 2000; Pasricha et al., 2017; Herrera et al., 2018; Pommier et al., 2020; Villalba et al., 2021). These assessment tools were chosen for their already proven test–retest and interitem reliability.

##### 4.2.2.1 Defining issues test assessment of moral reasoning

The DIT-2 is a device for activating moral schemas (to the extent that a person has developed them) and for assessing these schemas in terms of judgments. The DIT-2 (Rest, 1986a) presents five dilemmas and the participant rates and ranks items associated with these dilemmas in terms of their moral importance.

An example is the “Cancer (Story #4)” dilemma as follows: “Mrs. Bennet is 62 years old, and in the last phases of colon cancer. She is in terrible pain and asks the doctor to give her more pain-killer medicine. The doctor has given her the maximum safe dose already and is reluctant to increase the dosage because it would probably hasten her death. In a clear and rational mental state, Mrs. Bennet says that she realizes this, but she wants to end her suffering even if it means ending her life. Should the doctor give her an increased dosage?”

The participant is asked to rate the importance of questions pertaining to the scenario on a scale from one to five. As a point of clarification, the participant is asked to rate the importance of the question, not to answer the question itself. Three examples of the questions pertaining to the cancer scenario are as follows: (1) Is not the doctor obligated by the same laws as everybody else if giving an overdose would be the same as killing her? (2) Should only God decide when a person’s life should end? (3) Does the state have the right to force continued existence on those who do not want to live?

The answers to these types of questions, with the rating and ranking of 60 total statements pertaining to five scenarios, are used to determine the participant’s stage of moral reasoning (Center for the Study of Ethical Development, 2020).

##### 4.2.2.2 Compassion scale

The Compassion Scale (CS) is a 16-item assessment that has been shown to have strong psychometric properties representing a general factor of compassion for others. To validate the CS, Pommier et al. (2020) conducted six studies that operationalized compassion into four subscales representing greater kindness, common humanity, mindfulness, and lessened indifference. Support was found for construct validity, including divergent and convergent validity. Discriminant validity was established by findings that CS had small or nonsignificant correlations with social desirability, which is a key concern for this study. For the CS assessment, the participant is given a binary choice between true or false answers. Two example items from the CS scale are, “If I see someone going through a difficult time, I try to be caring toward that person,” and a reverse scored item, “I try to avoid people who are experiencing a lot of pain.”

In addition to the two assessments for moral reasoning and compassion, this study included direct measurements of a compassionate act and controlled for social desirability among participants. These measurements are described below.

##### 4.2.2.3 Compassionate act

To assess if a participant is more likely to behave compassionately beyond the self-reported assessments of moral reasoning (DIT-2) and compassion (CS), the following question was asked in the post assessment: Q—We recognize this experiment, and associated surveys were long and required a lot of thought. Each participant will receive $10.00 for their participation. If you would like, you can donate a portion of this amount to the United Nations Refugee Agency, but please do not feel any obligation to do so. Just click the appropriate box below and the survey will be complete. The multiple-choice answers to the compassionate act question include: (a) No donation at this time; (b) $1.00; (c) $3.00; (d) $5.00; and (e) $10.00. To avoid any uncomfortable circumstances around donation amounts or expectations, each participant was paid the full amount of $10.00 for their participation regardless of their pledge in the survey, and the participants were later informed that a donation exceeding the total sum of participant pledges ($214.00) was made on their behalf to the United Nations Refugee Agency.

##### 4.2.2.4 Control measurement for social desirability

Social desirability was measured and analyzed as a non-hypothesized control. This control was included as a measure to increase the validity of the findings through analysis of how high or low social desirability scores might influence the pre-post mean change of the dependent variables. The Marlow-Crowne Social Desirability Scale (M-C SDS) was used for the study because of its broad acceptance in terms of validity and reliability as well as its more than 13,000 citations (Crowne and Marlowe, 1960). To limit the number of total questions in the assessment, the short version, M-C 1(10) was used (Strahan and Gerbasi, 1972). A principal components analysis showed this version to have correlations with the original long version, M-C SDS, in the 80s and 90s among approximately 500 university students.

### 4.3 Results

The findings of the study are reported across both *The Displaced* VR experience and the *Kinoscope* VR experience in alignment with their associated hypotheses. As previously mentioned, the two VR film experiences were compared separately to understand whether the treatment experience influenced moral reasoning, and whether the control experience did not influence moral reasoning. The following sections include descriptive statistics, correlation analysis, and hypothesis tests using paired sample *t*-tests of pre-post mean scores (Gurley and Dagley, 2021).

#### 4.3.1 Descriptive statistics and correlation analysis

The mean age for participants was 18.83, with 53% male-identifying participants, 46% female-identifying participants, and 1% of participants who identified as “other.” The *education level* mean of 6.5 translates to students primarily in their second semester of their freshman year of college. The *political orientation* control variable is based on a possible score of 1–5, with five as the most conservative position and one as the most liberal position. The *political orientation* mean for this sample is 3.0. The *meaningless item* score was calculated by the Center for the Study of Ethical Development in their scoring of the DIT-2 assessments. The purpose of this score is to detect respondents who are trying to fake a high score. Five participants from the total study sample of 69 were purged from the analysis based partially on *meaningless item* scores >10. For the remainder of the participants included in *The Displaced* analysis, the *meaningless item* mean was 1.17. *Social desirability* was assessed with the 10-item Marlow-Crowne Social Desirability Scale (M-C SDS). A score of 10 represents the highest measured social desirability, and a score of 0 represents the lowest measured social desirability. The range for *The Displaced* sample on *social desirability* was 1–7, with *M* = 3.80. For the *act of compassion* analysis, participants’ range of donations was $0.00–$10.00, with *M* = $3.00 as represented by *compassion donation.* For the DIT-2 moral schema assessments, *Personal interest change* had a range of −32.00 to 34.00 and *M* = −5.53. *Maintaining norms change* had a range of −20.00 to 36.00 and *M* = 1.93. *Postconventional change* had a range of −22.00 to 30.00 and a *M* = 4.00 (Table 2).

**Table 2:** Descriptive statistics for *The Displaced* treatment experience.

*N* = 30	Range	Minimum	Maximum	Mean	Std. Deviation
Social desirability	6	1	7	3.80	1.73
Age	3.0	18.0	21.0	18.83	0.95
Conlib	4.0	1.0	5.0	3.00	1.08
Meaningless items	7.0	0.0	7.0	1.17	1.84
Compassion change	29	−12	17	0.73	6.24
Compassion donation	10	0	10	3.00	3.79
Personal interest change	66.00	−32.00	34.00	−5.53	14.89
Maintaining norms change	56.00	−20.00	36.00	1.93	14.37
Postconventional change	52.00	−22.00	30.00	4.00	13.51

For *Kinoscope* (*N* = 34), the mean *age* for participants was 19.29, with 47% male-identifying participants, 52% female-identifying participants, and 1% of participants who identified as “other.” The *education level M* = 6.5 translates to students primarily in their freshman year of college. The *political orientation* mean for this sample is 2.76. The *meaningless item* score for participants included in the *Kinoscope* analysis was *M* = 1.88. The range of “Cannot Decide” answers was zero to four, with *M* = 0.88. The range for the *Kinoscope* sample on *social desirability* was one to eight, with *M* = 4.32. For the *act of compassion* analysis, participants range of donations was $0.00 to $10.00, with *M* = $3.65 as represented by *compassion donation*. For the dependent variables assessed for their change from pre-treatment to post-treatment of the independent variable experience of *Kinoscope*, the descriptive statistics show the pre-post change. *Compassion change* had a range from −10 to 8 and *M* = −0.91. For the DIT-2 moral schema assessments, *personal interest change* had a range of −30.00 to 26.00 and *M* = −2.00. *Maintaining norms change* had a range of −18.00 to 26.00 and *M* = 1.12. *Postconventional change* had a range of −26.00 to 24.00 and *M* = 0.29.

Pearson correlation coefficients and associated significance at the *p* < 0.05^*^ and *p* < 0.01^**^ levels were used to examine the relationships between the dependent and control variables for both *The Displaced* treatment experience and the *Kinoscope* control experience. For *The Displaced*, there were no significant correlations between the control variables and dependent variables except for *education level* and p*ersonal interest change,* [*r*(30) = −0.383, *p* < 0.05^*^] and *education level* and *maintaining norms change* [*r*(30) = 0.525, *p* < 0.01^**^]. For *Kinoscope*, there were no significant correlations between the control variables and dependent variables.

#### 4.3.2 Hypothesis tests

The first step in hypotheses testing was to calculate pre-post mean change for all dependent variables in the experiment. Paired sample *t*-tests were then conducted for all hypothesized dependent variables for both *The Displaced* treatment experience and the *Kinoscope* control experience. The combined results of the mean change calculations and paired sample *t*-tests are shown in Table 3, with analysis of each specific hypothesis test to follow.

**Table 3:** Paired sample *t*-test of pre-post means for treatment and control experiences.

	Displaced pre/post assessment (*N* = 30)				Kinoscope pre/post assessment (*N* = 34)			
Dependent variable	Mean	Std. Deviation	*t* - statistic	Significance	Mean	Std. Deviation	*t* - statistic	Significance
Personal interest pretest	31.27	14.00			28.18	13.38		
Personal interest postest	25.73	16.35			26.18	14.81		
Personal interest change	−5.53	14.88	2.035	0.026*	−2.00	13.41	0.870	0.195
Maintaining norms pretest	32.07	12.49			27.94	13.43		
Maintaining norms postest	34.00	15.64			29.06	12.04		
Maintaining norms change	1.93	14.37	−0.737	0.234	1.12	12.45	−0.524	0.302
Postconventional pretest	31.73	15.15			36.12	16.97		
Postconventional postest	35.73	16.42			36.41	16.11		
Postconventional change	4.00	13.51	−1.621	0.058+	0.29	12.44	−0.138	0.446
Compassion pretest	80.93	6.92			84.26	6.85		
Compassion postest	81.67	7.62			83.35	7.77		
Compassion change	0.73	6.23	−0.644	0.262	−0.91	4.50	1.183	0.123

^*^Significant at the 0.05 level. ^+^Approaching significance at the 0.05 level.

##### 4.3.2.1 Hypothesis 1a

H1a was tested by first calculating the pre-post mean change scores for each of the moral schema variables including *personal interest change, maintaining norms change,* and *postconventional change*. The pre-post change in mean scores of the three moral reasoning schemas were *personal interest change* − 5.53 (*SD* = 14.89), *maintaining norms change* 1.93 (*SD* = 14.37), and *postconventional change* 4.00 (*SD* = 13.51). These results support the directional prediction of H1a with the participant’s *personal interest* transitioning lower and postconventional reasoning moving higher.

To determine the significance of the pre-post change in mean by moral schema for H1a, a paired sample *t*-test was conducted for each variable. For *personal interest* moral schema, there was a significant decrease between pre-test scores (*M* = 31.26, *SD* = 13.99) and post-test scores (*M* = 25.73, SD = 16.35); *t*(29) = 2.035, *p* = 0.026^*^. For the *maintaining norms* moral schema, there was not a significant change between pre-test scores (*M* = 32.06, *SD* = 12.48) and post-test scores (*M* = 34.00, *SD* = 15.64); *t*(29) = −0.737, *p* = 0.234. For the *postconventional change* moral schema, the increase between pre-test scores (*M* = 31.73, *SD* = 15.14) and post-test scores (*M* = 35.73, *SD* = 16.42); *t*(29) = −1.621, *p* = 0.058, was approaching significance. Because there was a significant decrease in the *personal interest* moral schema and the *postconventional change* moral schema scores for *The Displaced* VR experience were approaching significance, H1a is partially supported.

##### 4.3.2.2 Hypothesis 1b

H1b was tested using the same analytical procedure as H1a, by first calculating the pre-post mean change scores for each of the moral schema variables including *personal interest change*, *maintaining norms change,* and *postconventional change*. The pre-post change in mean scores of the three moral reasoning schemas were *personal interest change* − 2.00 (*SD* = 13.41), *maintaining norms change* 1.11 (*SD* = 12.45), and *postconventional change* 0.29 (*SD* = 12.44).

To determine the significance of the pre-post change in mean by moral schema for H1b, a paired sample *t*-test was conducted for each variable. For the *personal interest* moral schema, there was a not a significant decrease between pre-test scores (*M* = 28.18, *SD* = 13.38) and post-test scores (*M* = 26.18, *SD* = 14.81); *t*(33) = 0.870, *p* = 0.195. For the *maintaining norms* moral schema, there was not a significant change between pre-test scores (*M* = 27.94, *SD* = 13.43) and post-test scores (*M* = 29.06, *SD* = 12.04); *t*(33) = −0.524, *p* = 0.302. For the *postconventional* moral schema, there was not a significant increase between pre-test scores (*M* = 36.11, *SD* = 16.11) and post-test scores (*M* = 36.41, *SD* = 16.11); *t*(33) = −0.138, *p* = 0.446. Because there was not a significant change in any of the moral reasoning schema variables for *Kinoscope*, H1b is supported.

##### 4.3.2.3 Hypothesis 2a

H2a was tested by first calculating the pre-post mean change scores for compassion as measured by the CS (Pommier et al., 2020). To determine the significance of the pre-post change in mean for CS scores in analysis of H2a, a paired sample *t*-test was conducted. For the CS assessment of *The Displaced*, there was not a significant change between the pre-test scores (*M* = 80.93, *SD* = 6.92), and the post-test scores (*M* = 81.67, *SD* = 7.62); *t*(29) = −0.644, *p* = 0.262. The pre-post change in mean for the CS assessment was 0.733 (*SD* = 6.23). Based on the CS assessment, H2a was not supported.

##### 4.3.2.4 Hypothesis 2b

H2b was tested by first calculating the pre-post mean change scores for *compassion* as measured by the CS as described in the dependent variable measures section. To determine the significance of the pre-post change in mean for CS scores in analysis of H2b, a paired sample *t*-test was conducted. For the CS, there was a not a significant change between the pre-test scores (*M* = 84.26, *SD* = 6.85) and the post-test scores (*M* = 83.35, SD = 7.73); *t*(33) = 1.183, *p* = 0.123. The pre-post change in mean for the CS assessment was −0.91 (*SD* = 4.45). Based on the CS assessment, H2b was supported.

The calculated mean change, and pre-post paired sample *t*-test measured and analyzed significance of the CS items, which are a self-reported assessment administered as part of the survey. The study also measured a *compassionate act* as discussed in the methods section. The results of this measurement showed non-significant differences between *The Displaced* treatment experience and the *Kinoscope* control experience, increasing validity that H2a is not supported.

As shown in Table 4, the *Compassionate act* test results were opposite the prediction with donations after the *Kinoscope* experience (*M* = $3.65, *SD* = $4.59) being higher than donations after *The Displaced* experience (*M* = $3.00, *SD* = $3.79).

**Table 4:** Compassion donation comparison between treatment and control experiences.

	Displaced compassion donation M = $3.00 SD = $3.79		Kinoscope compassion donation M = $3.65 SD = $4.59	
Donation	Frequency	Percent	Frequency	Percent
$0.00	15	48.4	17	48.6
$1.00	2	6.5	3	8.6
$3.00	1	3.2	2	5.7
$5.00	7	22.6	1	2.9
$10.00	5	16.1	11	31.4
Total	30	96.8	34	97.1

##### 4.3.2.5 Hypotheses 3a and 3b

A one-tailed, paired-sample *t*-test analyzing the direct relationship between *The Displaced* treatment experience and the predicted mediating variable of *compassion* was not found to be significant, thus H3a was not supported. There was not a significant change between pre-test scores (*M* = 80.93, *SD* = 6.92) and post-test scores (*M* = 81.07, *SD* = 7.62); *t*(29) = 0.73, *p* = 0.262. H3b predicted no mediation influence from the control experience of *Kinoscope,* which was supported (*p* = 0.12), but not considered meaningful without support for H3a.

To further test if mediation occurred while comparing the results of *The Displaced* and *Kinoscope* in one model, simple mediation analysis was performed using PROCESS (Hayes, 2013). This analysis compared the results of *The Displaced* and *Kinoscope* as independent variables, the *personal interest* moral schema pre-post change as the dependent variable, and the CS assessment pre-post change as the mediator variable. This analysis confirmed that Hypothesis 3 was not supported [effect = −0.289, 95% C.I. (−2.33, 0.924)].

### 4.4 Experiment 1 discussion

The results of Experiment 1 found the hypotheses related to moral reasoning to be partially supported and the hypotheses related to compassion not supported. While participants who viewed *The Displaced* showed movement to higher stages of moral reasoning, their compassion scores did not significantly increase, therefore no mediation links between these variables were shown.

Participants who viewed *The Displaced* scored lower on the post-assessment of *personal interest*. Not only were the post-assessment mean scores of personal interest lower with *personal interest change* − 5.53 (S*D* = 14.89), *p* = 0.03^*^, but more than twice as many participants in the treatment group, 14 participants vs. six participants in the control group moved into the lower quartile of the sample. To illustrate the importance of this finding, one might imagine an educational scenario where a class of students is discussing alternative decisions that could be made by business or political leaders, concerning positions on societal issues such as labor practices, immigration, or sustainability. If there were significantly more students in the class taking a position with less self-interest, how might this influence the overall discussion and the individual development of the students? While not proposing an analysis or judgment of decision-making outcomes such as compassion or empathy, this possible scenario might encourage the development of broader and deeper decision-making skills through reasoning.

Most educators likely agree that helping students consider viewpoints beyond their personal interest is an important while ambitious goal, and that helping students develop deeper and broader forms of thought can sometimes be challenging. It involves increased understanding of teleological reasoning (Hunt and Vitell, 2006) to evaluate the consequences of various stakeholders, the desirability of the consequences, and the importance of the stakeholders to the decision maker. The *postconventional change* factor of the DIT-2 assessment tested this type of reasoning, as described by Kohlberg and Kramer (1969) as a universal-ethical-principle orientation, appealing to logical comprehensiveness and consistency, reciprocity, and equality of human rights. The study found that the influence of *The Displaced* was approaching significance with a *postconventional change* in mean of 4.00 (*SD* = 13.51), *p* = 0.058. While falling short of full support of H1a, this result does show transition from a narrower mindset of self-interest to a broader and deeper mindset of societal interest among participants.

The results of H2a were surprising, with *The Displaced* VR experience not having a significant influence on the pre-post CS assessment scores nor the *compassionate act*. For the CS assessment, there was not a significant change between the pre-test scores (*M* = 80.93, *SD* = 6.92) and the post-test scores (*M* = 81.67, *SD* = 7.62); *t*(29) = −0.644, *p* = 0.262. Therefore, H2a was not supported. The pre-post change in CS scores for *Kinoscope* viewers also did not show a significant increase, therefore H2b was supported with the CS assessment.

However, in the *compassionate act* assessment, which was conducted as an opportunity for the participants to donate all or a portion of their study compensation to the United Nations Refugee Agency, the results were opposite of the prediction for compassion, with *The Displaced* viewers donating less than the *Kinoscope* viewers. *The Displaced* viewers donated an average of $3.00 (*M* = $3.00, *SD* = $3.79), and the *Kinoscope* viewers donated an average of $3.65 (*M* = $3.65, *SD* = $4.59). This finding provides further evidence to disprove H2a, with *The Displaced* viewers not showing an increase in compassion and surprisingly mixed evidence with H2b supported by the self-assessment, but not supported by the actions of the participants.

## 5 Experiment 2

Experiment 2 is structured with similar methodology as Experiment 1, maintaining the same IV which is *The Displaced* VR film experience and the same control which is the *Kinoscope* VR film experience. As previously discussed, the consistency and repeatability of the VR film experiences allows for the testing of their possible influence on several constructs as DVs. For Experiment 2, the constructs of empathy and moral foundations were tested as the DVs.

### 5.1 Hypothesis development

**Figure 2:**
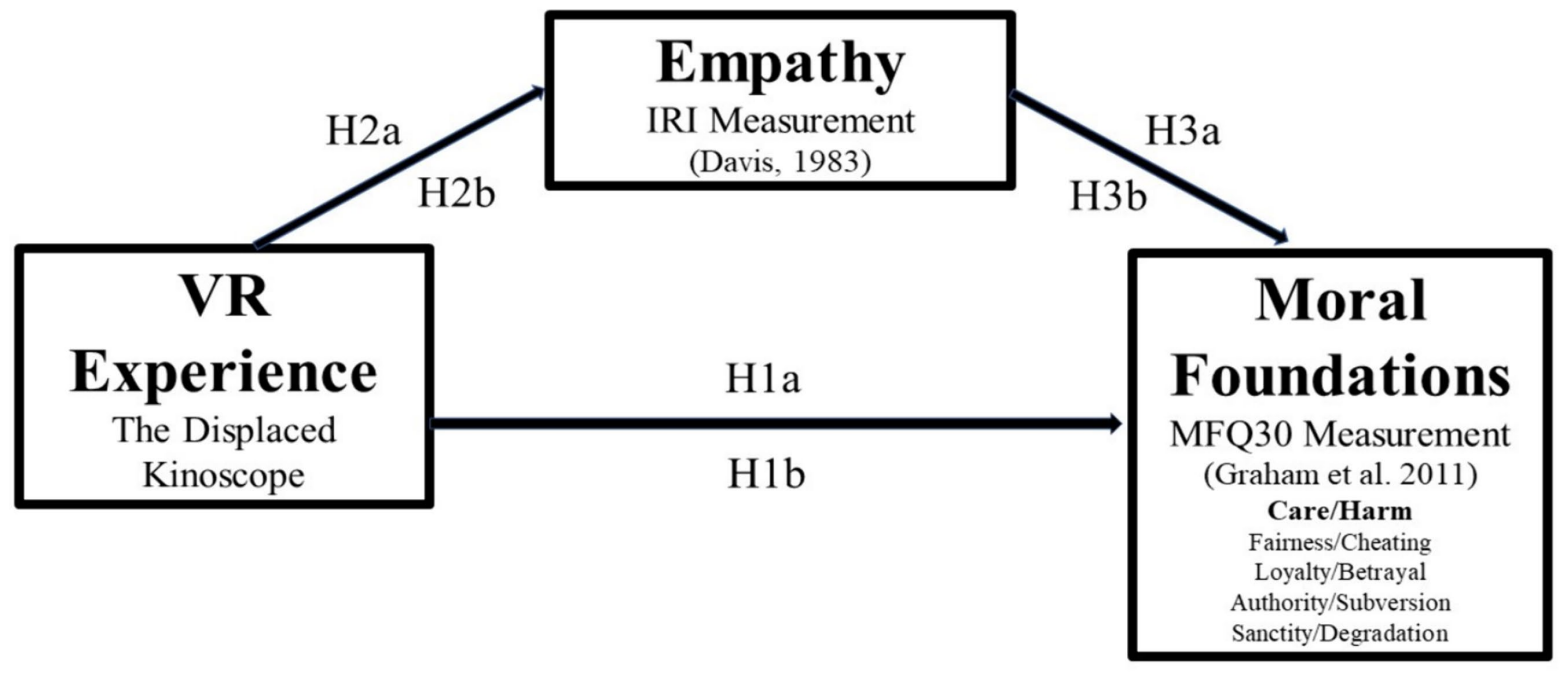
Hypothesized model for Experiment 2.

Hypothesis development for Experiment 2 is based on predicted links among the VR film experiences described in Section 4.2, measured change in empathy, and measured change in moral foundations as shown in Figure 2.

*The Displaced* was tested as the treatment experience (Ha), and *Kinoscope* was tested as a control experience (Hb). The first prediction of Experiment 2 is that participants empathy will increase after viewing *The Displaced.* In previous studies, the influence of VR experiences on empathy has been inconclusive (Seinfeld et al., 2018; Villalba et al., 2021; Sora-Domenjó, 2022), so it is recognized that with mixed results in previous studies, this prediction may be considered exploratory.

H1a: The Displaced *virtual reality experience will influence an increase the empathy.*

To control for factors outside the study that may have contributed to the results of H1a, the influence of *Kinoscope* was tested as an experience not expected to influence empathy. The logic of this approach is that if the participant’s empathy increases in the H1a group while the empathy of participants in the H1b group does not, an assumption can be made that outside influences were not the cause of this transition because they would have influenced both groups. Therefore, H1b predicts that *Kinoscope* will not influence empathy.

H1b: *The* Kinoscope *virtual reality experience will not influence an increase in empathy.*

The prediction of the second set of hypotheses investigates if *The Displaced* VR experience will influence the Care/harm factor of moral foundations. This prediction may be considered exploratory with no known theoretical history of moral foundations being tested with VR experiences as independent variables at the time of Experiment 2. All five factors of *moral foundations theory* were tested, including Care/harm, Fairness/cheating, Loyalty/betrayal, Authority/subversion, Sanctity/degradation, but only the Care/harm factor is hypothesized based on the content of *The Displaced* VR film experience.

H2a*: The Care/harm factor of moral foundations will be higher after the participant is immersed into* The Displaced *VR experience.*

In alignment with the methodology used for H1a and H1b, the second prediction is tested among two experimental groups within the same participant sample, to control for outside factors which might have influenced results during the three-week period, from pre-assessment to treatment to post-assessment.

H2b: *The Care/harm factor of moral foundations theory will not be higher after the participant is immersed into The* Kinoscope *VR experience.*

Based on the results of the first and second predictions, statistical analysis determines the possible mediation influence of empathy on the possible increase in Care/harm for each of the experimental groups.

H3a: *Empathy will partially mediate the relationship between* The Displaced *VR experience and an increase in Care/harm.*

H3b: *Empathy will not mediate the relationship between the* Kinoscope *VR experience and an increase in Care/harm.*

It is important to note that the purpose of the study and the associated hypotheses are not proposed to compare the level of effects between the VR experiences. The study is designed to test if one experience, *The Displaced,* will influence empathy and/or Care/harm, whereas another experience, *Kinoscope* will not influence empathy and/or Care/harm.

### 5.2 Methods

#### 5.2.1 Participants and procedure

Experiment 2 was conducted at the same laboratory as Experiment 1 with a sample of 44 participants who completed the experiment, including 25 in *The Displaced* VR experience treatment group, and 19 in the *Kinoscope* control group.

Participants were undergraduate students enrolled in marketing and entrepreneurial business classes at a university in the mountain states of the United States. The study was introduced to the students via zoom conference calls with follow-up emails for pre-post surveys via Qualtrics. The purpose of the study was not communicated, but the virtual reality component of the experiment was described.

In phase 1 of the experiment, participants completed a questionnaire consisting of three sections. The first section included all demographic information necessary for the control variables. The second section included the Interpersonal Reactivity Index (IRI) to measure empathy. The third section included the Moral Foundations Questionnaire (MFQ30) which measures the participants’ moral decision-making processes. Phase 2 was conducted approximately 2 weeks after phase one. This phase began as the participants arrived at the laboratory location. Upon arrival, participants confirmed phase one data had been collected; then each participant was given further instructions by one of the researchers informing the participant how to use the VR headset with the randomly assigned experience queued. Upon completion of the VR experience, phase 2 continued with the post-assessment questionnaire, including sections two and three for empathy and moral foundations.

#### 5.2.2 Dependent variables

##### 5.2.2.1 Moral foundations questionnaire

Moral Foundations was measured using the MFQ30, which has been shown to provide an effective measure of moral foundations with proven convergent and divergent validity (Haidt, 2001, 2012a,b; Graham et al., 2013; Andersen et al., 2015). It utilizes 30 statements evaluated on a six-point Likert scale with 15 statements evaluated where 0 = not at all relevant and 5 = extremely relevant and the other 15 statements are evaluated with 0 = strongly disagree and 5 = strongly agree. There are two check questions with 32 total items to complete. The analysis of the MFQ30 of Graham et al. (2011) includes exploratory factor analysis (EFA), test–retest reliability with 123 participants, confirmatory factor analysis (CFA), relations to other scales to determine convergent and discriminant validity, cross-cultural differences with participants from South Asia, East Asia, United States, United Kingdom, Western Europe, and Canada.

##### 5.2.2.2 Interpersonal reactivity index—empathy assessment

Empathy was measured using the IRI (Davis, 1983). The IRI measures individual differences in empathy through placing the participant into the point of view of others. It includes 28-items answered on a five-point Likert scale ranging from “Does not describe me well” to “Describes me very well.” The measure has four subscales, each made up of seven different items. These subscales are defined by Davis (1983) as: (1) Perspective Taking—the tendency to spontaneously adopt the psychological point of view of others, (2) Fantasy—taps respondents’ tendencies to transpose themselves imaginatively into the feelings and actions of fictitious characters in books, movies, and plays, (3) Empathic Concern—assesses “other-oriented” feelings of sympathy and concern for unfortunate others, and (4) Personal Distress—measures “self-oriented” feelings of personal anxiety and unease in tense interpersonal settings.

### 5.3 Results

A similar analysis was conducted for Experiment 2 as for Experiment 1 with a summary of results described below. *The Displaced* treatment sample included 25 participants with a mean age of 20.24. There were 32% male-identifying participants and 68% female-identifying participants. For ethnicity, 76% of participants identified as White, 16% Hispanic, and 4% Asian. Descriptive statistics for each of the DVs is shown in Table 5. These results are grouped by pre-test and post-test scores for each factor of the construct measurements for empathy (IRI) and moral foundations (MFQ30). Statistical analysis showed no meaningful correlations between demographic controls and pre-post change in DVs.

**Table 5:** Descriptive statistics for *The Displaced* treatment experience dependent variables.

Construct measures	Pre/Post factors	*N*	Mean	Std. Deviation	Variance	Range	Minimum	Maximum
Empathy - IRI	Fantasy1	25	18.96	4.97	24.71	19.00	8.00	27.00
Empathy - IRI	Fantasy2	25	19.44	4.75	22.59	18.00	10.00	28.00
Empathy - IRI	Empathy1	25	18.96	2.73	7.46	11.00	14.00	25.00
Empathy - IRI	Empathy2	25	19.08	2.93	8.58	12.00	13.00	25.00
Empathy - IRI	Perspective1	25	18.12	4.28	18.28	18.00	9.00	27.00
Empathy - IRI	Perspective2	25	18.28	4.05	16.38	18.00	9.00	27.00
Empathy - IRI	Distress1	25	13.28	4.22	17.79	16.00	6.00	22.00
Empathy - IRI	Distress2	25	12.96	3.82	14.62	16.00	5.00	21.00
Empathy - IRI	EC/PT1	25	37.20	6.18	38.17	23.00	28.00	51.00
Empathy - IRI	EC/PT2	25	37.36	6.02	36.24	20.00	28.00	48.00
Empathy - IRI	ALLEMP1	25	69.32	9.75	95.14	36.00	55.00	91.00
Empathy - IRI	ALLEMP2	25	69.76	9.42	88.69	28.00	59.00	87.00
MFQ-30	Care1	25	21.20	4.47	20.00	20.00	10.00	30.00
MFQ-30	Care2	25	22.12	3.94	15.53	15.00	14.00	29.00
MFQ-30	Fairness1	25	22.32	3.30	10.89	14.00	14.00	28.00
MFQ-30	Fairness2	25	22.72	3.30	10.88	15.00	14.00	29.00
MFQ-30	Loyalty1	25	15.40	4.62	21.33	19.00	4.00	23.00
MFQ-30	Loyalty2	25	15.08	5.29	27.99	21.00	4.00	25.00
MFQ-30	Authority1	25	16.56	4.31	18.59	16.00	9.00	25.00
MFQ-30	Authority2	25	16.44	4.20	17.67	15.00	8.00	23.00
MFQ-30	Sanctity1	25	16.28	6.24	38.96	25.00	3.00	28.00
MFQ-30	Sanctity2	25	16.16	6.82	46.47	27.00	3.00	30.00

To test the hypotheses for *The Displaced* as the treatment variable, a pre-post mean change for the factors of moral foundations and empathy were calculated. A paired sample *t*-test was then used to analyze the change in participant’s mean scores from before and after the VR experience. The results of this analysis are shown in Table 6.

**Table 6:** Pre-post paired sample *t*-test of empathy and moral foundations for *The Displaced.*

Dependent variables		Pre/Post mean change	Std. Deviation	Std. Error mean	95% confidence interval				Significance
Construct measures	Pre1/Post2 factors				Lower	Upper	*t*	df	One-sided *p* value
Empathy - IRI	Fantasy1 - Fantasy2	0.48	2.55	0.51	−1.53	0.57	−0.94	24.00	0.18
Empathy - IRI	Empathy1 - Empathy2	0.12	2.13	0.43	−1.00	0.76	−0.28	24.00	0.39
Empathy - IRI	Perspective1 - Perspective2	0.16	2.30	0.46	−1.11	0.79	−0.35	24.00	0.37
Empathy - IRI	Distress1 - Distress2	−0.32	2.04	0.41	−0.52	1.16	0.79	24.00	0.22
Empathy - IRI	EC/PT1 - EC/PT2	0.16	3.46	0.69	−1.59	1.27	−0.23	24.00	0.41
Empathy - IRI	ALLEMP1 - ALLEMP2	0.44	5.70	1.14	−2.79	1.91	−0.39	24.00	0.35
MFQ-30	Care1 - Care2	0.92	2.52	0.50	−1.96	0.12	−1.83	24.00	0.04*
MFQ-30	Fairness1 - Fairness2	0.40	2.89	0.58	−1.59	0.79	−0.69	24.00	0.25
MFQ-30	Loyalty1 - Loyalty2	−0.32	2.30	0.46	−0.63	1.27	0.69	24.00	0.25
MFQ-30	Authority1 - Authority2	−0.12	3.11	0.62	−1.17	1.41	0.19	24.00	0.42
MFQ-30	Sanctity1 - Sanctity2	−0.12	3.02	0.60	−1.13	1.37	0.20	24.00	0.42

^*^Significant at the 0.05 level.

For *The Displaced*, Hypothesis 1a was supported with the mean of the moral foundations Care/harm factor increasing between pre-test scores (M = 21.20, SD = 4.47) and post-test scores (M = 22.12, SD = 3.94); *t*(24) = −1.83, *p* = 0.04^*^. This result is unique and in contrast to all the other DVs measured with *p* values above 0.22, except for Empathy/Fantasy at *p* = 0.18. The second hypothesis for *The Displaced*, H2a predicted that empathy would be increased after viewing the VR experience. This hypothesis was not supported with the overall empathy score increasing below significance from pre-test scores (M = 69.32, SD = 9.75) and post-test scores (M = 69.76, SD = 9.42); *t*(24) = −0.39, *p* = 0.35. Because *The Displaced* did not significantly influence empathy directly, mediation could also not occur. Therefore, hypothesis 3a was not supported.

The sample for *Kinoscope* included 19 participants with a mean age of 19.74. There were 32% male-identifying participants and 68% female-identifying participants. For ethnicity, 85% of participants identified at White, 5% Hispanic, 5% Asian, and 5% Middle Eastern. Descriptive statistics for the *Kinoscope* sample on each of the dependent variables is shown in Table 7. These results are grouped by pre-test and post-test scores for each factor of the construct measurements for empathy (IRI) and moral foundations (MFQ30). Statistical analysis showed no significant correlations between demographic controls and pre-post change in DVs.

**Table 7:** Descriptive statistics for *Kinoscope* control experience dependent variables.

Construct measures	Pre/Post factors	*N*	Mean	Std. Deviation	Variance	Range	Minimum	Maximum
Empathy - IRI	Fantasy1	19	19.11	4.27	18.21	16.00	12.00	28.00
Empathy - IRI	Fantasy2	19	20.42	5.20	27.04	17.00	11.00	28.00
Empathy - IRI	Empathy1	19	19.47	2.37	5.60	7.00	16.00	23.00
Empathy - IRI	Empathy2	19	20.32	2.81	7.90	10.00	16.00	26.00
Empathy - IRI	Perspective1	19	18.16	3.40	11.59	11.00	13.00	24.00
Empathy - IRI	Perspective2	19	19.26	2.08	4.32	7.00	16.00	23.00
Empathy - IRI	Distress1	19	12.53	3.42	11.71	16.00	2.00	18.00
Empathy - IRI	Distress2	19	12.05	4.06	16.50	19.00	1.00	20.00
Empathy - IRI	EC/PT1	19	37.79	4.02	16.18	14.00	29.00	43.00
Empathy - IRI	EC/PT2	19	39.58	3.12	9.70	10.00	34.00	44.00
Empathy - IRI	ALLEMP1	19	69.26	7.42	55.09	25.00	56.00	81.00
Empathy - IRI	ALLEMP2	19	72.05	8.35	69.72	28.00	60.00	88.00
MFQ-30	Care1	19	22.42	3.20	10.26	12.00	15.00	27.00
MFQ-30	Care2	19	22.05	2.92	8.50	14.00	16.00	30.00
MFQ-30	Fairness1	19	21.58	3.60	12.92	16.00	14.00	30.00
MFQ-30	Fairness2	19	22.16	3.08	9.47	11.00	18.00	29.00
MFQ-30	Loyalty1	19	15.42	5.59	31.26	20.00	6.00	26.00
MFQ-30	Loyalty2	19	15.32	5.96	35.56	20.00	5.00	25.00
MFQ-30	Authority1	19	16.37	5.20	27.02	19.00	5.00	24.00
MFQ-30	Authority2	19	16.11	6.41	41.10	22.00	5.00	27.00
MFQ-30	Sanctity1	19	15.11	6.03	36.32	18.00	6.00	24.00
MFQ-30	Sanctity2	19	14.63	6.09	37.14	21.00	6.00	27.00

To test the hypotheses for *Kinoscope* as the control experience, a pre-post mean change for the factors of moral foundations and empathy were calculated. A paired sample *t*-test was then used to analyze the change in participant’s mean scores from before and after the VR experience. The results of this analysis are shown in Table 8.

**Table 8:** Pre-post comparison of empathy and moral foundations for *Kinoscope.*

Dependent variables		Pre/Post mean Change	Std. Deviation	Std. Error mean	95% confidence interval				Significance
Construct measures	Pre/Post items				Lower	Upper	*t*	df	One-sided *p* value
Empathy - IRI	Fantasy1 - Fantasy2	1.316	2.083	0.478	−2.32	−0.312	−2.753	18	0.007^**^
Empathy - IRI	Empathy1 - Empathy2	0.842	1.864	0.428	−1.74	0.056	−1.969	18	0.032^*^
Empathy - IRI	Perspective1 - Perspective2	1.105	2.826	0.648	−2.468	0.257	−1.705	18	0.053
Empathy - IRI	Distress1 - Distress2	−0.474	1.837	0.421	−0.412	1.359	1.124	18	0.138
Empathy - IRI	EC/PT1 - EC/PT2	1.789	3.537	0.811	−3.494	−0.085	−2.205	18	0.020^*^
Empathy - IRI	ALLEMP1 - ALLEMP2	2.789	5.277	1.211	−5.333	−0.246	−2.304	18	0.017^*^
MFQ-30	Care1 - Care2	−0.368	2.216	0.508	−0.7	1.437	0.725	18	0.239
MFQ-30	Fairness1 - Fairness2	0.579	1.924	0.441	−1.506	0.348	−1.312	18	0.103
MFQ-30	Loyalty1 - Loyalty2	−0.105	2.942	0.675	−1.313	1.523	0.156	18	0.439
MFQ-30	Authority1 - Authority2	−0.263	3.572	0.82	−1.459	1.985	0.321	18	0.376
MFQ-30	Sanctity1 - Sanctity2	−0.474	3.289	0.755	−1.112	2.059	0.628	18	0.269

^*^Significant at the 0.05 level.

^**^Significant at the 0.01 level.

Hypothesis 1b predicted *Kinoscope* would not influence the Care/harm factor of moral foundations, providing a control experience in contrast to *The Displaced* treatment experience among the same random sample of participants and within the same window of time. The results for the Care/harm factor of moral foundations were pre-test scores (M = 22.42, SD = 3.20) and post-test scores (M = 22.05, SD = 2.92); *t*(18) = 0.725, *p* = 0.239. Hypothesis 2b was supported confirming *The Displaced* VR experience influencing the Care/harm factor of moral foundations while the *Kinoscope* VR experience did not influence the Care/harm factor of moral foundations.

Hypothesis 2b predicted Kinoscope would not influence empathy based on the VR experience selection process described in sections 4.1 and 4.2. The results however were surprising with empathetic concern and fantasy factors of empathy showing significant pre-post change in mean. Hypothesis 2b was not supported, with the findings adding to the mixed results from previous studies investigating the influence of VR experiences on empathy.

While overall empathy, comprising the sum of means for all four factors, was significantly increased in the pre-test scores for *Kinoscope*, the Care/harm factor was not significantly influenced. Therefore, no mediation occurred and Hypothesis 3b was supported.

### 5.4 Experiment 2 discussion

An increase in the Care/harm factor of moral foundations among participants who viewed *The Displaced* (Pre-test mean of 21.20 increasing to Post-test mean of 22.12, *p* = 0.04^*^) supports H1a, and provides evidence that the film influenced this pre-post change. While the results are limited to this one experiment, the authors propose that this finding supports further investigation of how VR experiences might influence the Harm/care factor of moral foundations along with the other foundations of loyalty, authority, sanctity, and fairness.

One implication for the *Displaced* influencing Care/harm is that the VR film might be incorporated with other learnings as a pedagogical tool to stimulate understanding and discussion for how political conflict and war can affect individual lives. Other VR experiences might also be utilized to show the importance of loyalty for an organization’s success, or authority in following procedures in an urgent care facility. Perhaps these findings can stimulate further research to build VR experiences and applications as teaching tools that may be applied in difficult and unique situations that are not possible to emulate in safe and secure environments.

It is surprising that the results showed a significant increase in empathy among the viewers of *Kinoscope*, and did not indicate a significant increase in empathy among participants viewing *The Displaced*. One possible explanation for this lack of support for H1b and H2b is that the IRI (Davis, 1983) assessment is not measuring what many scholars and advocates of VR define as empathy. The two factors in the empathy scale influencing the results for *Kinoscope* were Fantasy and Empathetic Concern. With the *Kinoscope* film documenting the history of cinema; could it be the content of the film, which included scenes of fictional stories, increased the participant’s fantasy factor scores of empathy? While intuitive explanations can be hypothesized for these results, there is not a clear explanation why *The Displaced* did not produce significant influence in any of the four factors of the IRI scale. The findings of significance for the Fantasy and Empathetic Concern factors further support the need for a clearer definition of the empathy construct in the literature on the VR-empathy model, particularly as measured by the IRI.

In their study of how a VR experience about homelessness influences the behavioral act of signing a petition to help the homeless, and also self-reported empathy as measured by the IRI, Herrera et al. (2018) found support for the VR experience influencing the compassionate act of signing the petition, but not for the VR experience influencing an increase in empathy. Several scholars have pointed out the need for more research and clarity of definitions (Bloom, 2017) and have challenged many widely held assumptions about human empathy. The findings in Experiment 2 support a need for this further investigation.

## 6 Overall discussion and limitations

The findings from the overall study, encompassing all four constructs measured in both Experiment 1 and Experiment 2, reveal some expected results, some surprising results and also raise several questions. In addition to the discussion around mixed results for empathy, why did participants move higher on the stages of moral reasoning without scoring higher on compassion? For the same random sample of participants, why would viewers of *The Displaced* score higher on the moral foundations factor of Care/harm and then also move to higher stages of moral reasoning. Why do the results point toward a possible relationship between moral reasoning and the Care/harm factor of moral foundations, but without a relationship between the constructs of empathy and compassion? One possibility might stem from the content of the scale items used in the assessments for each construct.

In their article, the Interplay between Absolute Language and Moral Reasoning on Endorsement of Moral Foundations, Blankenship et al. (2021) suspected that the language of scale items may have influenced the results of their study. They point toward a majority of topics used to assess moral reasoning to be closely related to the Care/harm factor of moral foundations with topics or issues including killing, euthanasia, betrayal and deception. They state that their research highlights an issue with measures of moral reasoning with many scale items used tapping into the moral foundation of Care/harm. Whether this is an issue or not can be debated, but it is something to recognize not only for the assessments of moral reasoning and moral foundations, but also for the assessments of empathy and compassion.

In addressing the question of why participants moved to higher stages of moral reasoning while not scoring higher on the compassion scale, one might suggest a similar conclusion as the one offered above, that the language of the scales influenced the results. While this is certainly a possibility, with more research needed in this area, it does not offer explanation for the results of the direct measurement of compassion where participants viewing *Kinoscope*, the VR film about the history of cinema, donated more money to the United Nations Refugee Agency than participants who viewed *The Displaced*, which depicted the dystopia of refugee children? Could it be that moral reasoning and compassion are truly not linked? Can one move from stages of personal interest to stages of community and societal interests without compassion being a component of this shift?

Another possible reason for the results not linking compassion with moral reasoning may stem from the challenges of operationalizing the constructs, including the possibility of confounding variables. The participants not showing an increase in the compassionate act measurement could potentially be explained by the influence of moral circles. In a study on the influence of moral circles, Baron and Miller (2000) found participants from the United States and India were influenced by how distant a potential recipient of a bone marrow donation was to the donor. Both groups became less willing to donate as physical distance from the recipient increased. Their study suggests the motivation to act compassionately might be affected by the distance of the beneficiary of the action. In the case of our study, the children depicted in *The Displaced* were in countries thousands of miles from the participants. The influence of moral circles may be an important factor when a participant decides who is eligible to receive the benefits of a prosocial action.

With significant links shown between a virtual reality experience and the theories of moral reasoning (Kohlberg and Kramer, 1969) and moral foundations (Graham et al., 2013), we hope to have opened a door for additional research on these possible relationships. What other VR experiences can be tested that may possibly advance a viewer to higher or lower stages of moral reasoning? With the capabilities of VR allowing researchers to control the independent variables, we can test previously held beliefs about our assessments of empathy, compassion, moral foundations and moral reasoning, comparing the results across constructs and evaluating the validity and consistency of measurements like the IRI, the CS, the MFQ30, and the DIT-2 for use in specific circumstances. Researchers incorporating neuroscience and bioinformatic technologies are already advancing our understanding of VR’s influence on human morality, and we propose the addition of these constructs and assessments to be tested in future studies utilizing these techniques.

This study is limited in scope to the constructs measured in relation to the specific VR experiences. It does not seek to answer questions such as the influence of VR vs. traditional media, which have already been documented in several studies with mixed results. Archer and Finger (2018) found that immersive formats resulted in stronger empathic responses than traditional media, with a higher probability of participants taking part in political or social actions. Research conducted at Oxford University compared the prosocial impact of conventional and immersive media finding that target-specific VR formats have a bigger influence on users (Van Loon et al., 2018). In a study comparing effects on empathy between participants consuming the content as either a written script, two-dimensional screened video or 360-degree, three-dimensional immersive virtual reality experience, Steinfeld (2020) showed no correlation between the method of content consumption and participant’s empathetic reaction.

The methodology and findings of our study are limited by the demographics of the sample, which only included university students from the United States. There can be no claims of generalizability and we encourage similar studies to be conducted across cultures. Additional limitations include the self-reported assessments utilized for dependent variables, the process of choosing which VR experiences to test, and the lack of previous studies linking VR to compassion, moral reasoning, and moral foundations.

Larger studies across geo-political groups, levels of education, cultures, religions, and several other demographics will provide increased validity, reliability and insights to possibly predict and drive specific outcomes. Incorporating VR into experiential education programs might possibly help our students develop higher forms of moral reasoning to address the collective challenges we face as a global community. We do not propose that higher stages of moral reasoning are right while lower stages are wrong, but we do propose the development of more complex decision-making skills may help students move beyond singular and dichotomous levels of thought and decision-making tendencies involving primarily personal interest.

This research examines how one VR experience might influence four of the primary constructs commonly studied and discussed in the literature of moral psychology, with the findings limited to this purpose, but it addresses additional questions as well. The first is to provide insight to better understand why empathy has not been clearly and consistently linked to the experiences of VR in quantitative studies (Seinfeld et al., 2018; Villalba et al., 2021; Sora-Domenjó, 2022). This includes the debate over construct definitions of empathy and compassion, (Cuff et al., 2016; Bloom, 2017; Hall and Schwartz, 2019). Still further questions arise on the validity of our assessments in general, and how the language used in these assessments may contribute to our results, (Blankenship et al., 2021). But perhaps the most important question pertains to our understanding of how VR experiences can possibly contribute to pro-social behavior.

While it is important to increase our understanding of how links between VR experiences and specific constructs might occur, it is even more important to understand how we can use these experiences to drive specific thoughts, behaviors and outcomes. How can VR be incorporated into experiential learning programs that might help educators advance the moral reasoning, moral foundations, empathy and compassion of their students? This study takes small, but hopefully useful steps toward addressing these questions and objectives.

## Data availability statement

The raw data supporting the conclusions of this article will be made available by the authors, without undue reservation.

## Ethics statement

The studies involving humans were approved by University of Denver Internal Review Board. The studies were conducted in accordance with the local legislation and institutional requirements. The participants provided their written informed consent to participate in this study.

## Author contributions

DD: Conceptualization, Data curation, Formal analysis, Funding acquisition, Investigation, Methodology, Project administration, Resources, Software, Supervision, Validation, Visualization, Writing – original draft, Writing – review & editing. PM: Data curation, Investigation, Methodology, Resources, Validation, Writing – original draft, Writing – review & editing. DC: Data curation, Formal analysis, Investigation, Validation, Writing – review & editing. DW: Methodology, Project administration, Supervision, Validation, Writing – review & editing.
